# Development of a rapid homogeneous immunoassay for detection of rotavirus in stool samples

**DOI:** 10.3389/fpubh.2022.975720

**Published:** 2022-08-04

**Authors:** Ye Wang, Yuling Zheng, Yan Li, Shengwei Zhang, Xin Wang, Huijun Zong, Wenhua Huang, Decong Kong, Yongqiang Jiang, Peng Liu, Qingyu Lv, Hua Jiang

**Affiliations:** ^1^College of Life Science and Technology, Beijing University of Chemical Technology, Beijing, China; ^2^State Key Laboratory of Pathogen and Biosecurity, Institute of Microbiology and Epidemiology, Academy of Military Medical Sciences (AMMS), Beijing, China; ^3^The Fifth Medical Center of PLA General Hospital, Beijing, China; ^4^Department of Clinical Laboratory, Dongfang Hospital, Beijing University of Chinese Medicine, Beijing, China

**Keywords:** diarrhea, rotavirus, detection, AlphaLISA, homogeneous assay

## Abstract

Rotavirus is the main pathogen causing acute viral gastroenteritis. Accurate and rapid diagnosis of rotavirus infection is important to determine appropriate treatment, prevention of unnecessary antibiotics use and control of infection spread. In this study, we established a rapid, accurate, and sensitive amplified luminescent proximity homogeneous assay linked immunosorbent assay (AlphaLISA) for detecting rotavirus and evaluated its efficacy in human stool samples. Our results demonstrated that the sensitivity of AlphaLISA (5^−8^) significantly exceeded that of the immunochromatographic assay (ICA, 5^−4^) for rotavirus antigen detection. The intra-assay and inter-assay coefficients of variation were 2.99–3.85% and 5.27–6.51%, respectively. Furthermore, AlphaLISA was specific for rotavirus and did not cross-react with other common diarrhea viruses. AlphaLISA and real-time reverse transcription polymerase chain reaction (RT-qPCR, which is considered a gold standard for detecting diarrhea viruses) tests showed consistent results on 235 stool samples, with an overall consistency rate of 97.87% and a kappa value of 0.894 (*P* < 0.001). The overall consistency rate of ICA compared with RT-qPCR was 95.74%. AlphaLISA showed better consistency with RT-qPCR than the routinely used ICA for rotavirus detection in stool samples. The AlphaLISA method can be used in clinical practice for the rapid, accurate, and sensitive detection of rotavirus infection.

## Introduction

Acute gastroenteritis (AGE) is a relatively common infectious disease that affects hundreds of millions of people worldwide every year, especially in low-income countries. It is one of the leading causes of illness and death in children under 5 years of age (1, 2). Rotavirus is the major cause of acute viral gastroenteritis in infants and young children, which is transmitted primarily *via* the fecal-oral route (3). Watery diarrhea, vomiting, headache, fever, and stomachic abdominal cramps are all clinical symptoms of rotavirus illness (4). Rotavirus infection can cause asymptomatic or mild diarrhea in adults, but immunocompromised individuals are particularly susceptible to infection and can suffer from severe diarrhea (5). Patients with gastroenteritis are primarily treated with oral drugs or intravenous fluids. Viral gastroenteritis is usually not treated with antibiotics (6), so accurate and rapid identification of gastroenteritis pathogens could help reduce unnecessary antibiotic use.

Numerous techniques can be used for rotavirus detection, including traditional detection methods, immunological detection methods, and molecular biological detection methods. Traditional detection methods such as virus isolation in cell culture, electron microscopy, and serological tests are difficult and lengthy to operate (7). Immunological detection methods include enzyme-linked immunosorbent assay (ELISA) technology and immunochromatographic assay (ICA) to detect pathogens through the specific binding of antibodies and antigens. ELISA requires multiple washing steps to remove nonspecifically bound reactants, which is time-consuming (8). The immunochromatographic assay can give detection results in a short time, but has low sensitivity (9). Molecular biological methods such as real-time quantitative reverse transcription polymerase chain reaction (RT-qPCR) are highly sensitive and specific, but they require specialized techniques and equipment and take a lot of time, which is not conducive to rapid detection and large-scale screening (10).

This study developed a rotavirus detection method based on the amplified luminescent proximity homogeneous assay linked immunosorbent assay (AlphaLISA), which primarily depends on the interaction between donor microspheres and acceptor microspheres. The surface of donor microspheres has been labeled with streptavidin, which can capture biotinylated antibodies. The acceptor microspheres are then conjugated with detection antibodies. The two microspheres were close when the test antigen was bound to the specific antibody. The photosensitizer on the donor emitted singlet oxygen molecules if irradiated by a 680 nm laser, and the singlet oxygen molecules proliferate and reach the surface of acceptor beads in the proximity of 200 nm. This triggers a chemical reaction and generates a chemiluminescence signal at 615 nm on the surface of the acceptor beads (11). The AlphaLISA has many advantages over conventional detection methods: it is easy to operate, highly sensitive, fast, uses less volume of sample, and has become a highly accurate *in-vitro* diagnostic tool. In this study, we evaluate this system for rotavirus detection in stool and compare the efficacy of AlphaLISA with conventional detection methods.

## Materials and methods

### Stool samples

Two hundred and thirty five stool samples were collected from patients with symptoms of acute gastroenteritis from the Fifth Medical Center of PLA General Hospital and Dongfang Hospital in Beijing, between December 2019 and June 2022. Patient ages ranged from 7 days to 91 years (average, 42.9 years); 26 samples (11.1%) were from patients under 5 years of age and 54 samples (23.0%) were from patients who were 65 years of age or older. The fresh stool samples were aliquoted and frozen immediately at −80°C until they were used for the comparative tests (AlphaLISA, RT-qPCR and ICA). This study was approved by the Institutional Review Board (IRB) of the Fifth Medical Center of PLA General Hospital (reference no. ky-2019-1-4) and Dongfang Hospital (reference no. JDF-IRB-2020003501). All samples were obtained with the patient's consent.

### Antigen, antibody, and reagent

Rotavirus antigen (Simian rotavirus SA11) inactivated using gamma irradiation was purchased from Microbix (Toronto, Canada). Rotavirus antibodies 10R-30C and 10R-30E were purchased from Fitzgerald (North Acton, MA, USA). Unconjugated Eu-acceptor beads, streptavidin-coated donor beads, 1/2 AreaPlateTM-96 well plate, and 10 × AlphaLISA immunoassay buffer were purchased from PerkinElmer (Waltham, MA, USA). EZ-Link^®^ Sulfo-NHS-LC-Biotinylation Kit was purchased from Thermo Fisher Scientific (Waltham, MA, USA). NaBH_3_CN and carboxymethoxylamine were purchased from Sigma (St. Louis, MO, USA).

### AlphaLISA test

Three microliter biotin (10 mM) was added to 100 μg of the rotavirus antibody (10R-30E) solution. After 1 h of incubation at room temperature, the excess biotin was removed by a Zeba Spin Desalting Column. Conjugation of the rotavirus antibody (10R-30C) to acceptor beads was performed according to the manufacturer's instructions as previously described (12).

Five milligram stool samples were weighed and placed in 1.5 ml centrifuge tubes 0. 1 ml PBS (pH 7.4) was added to make a 0.5% (w/v) suspension. This suspension was mixed by vortex and centrifuged at 2,500 g for 5 min. The supernatant was collected for the AlphaLISA testing.

AlphaLISA was performed in a white 1/2 AreaPlate™-96. Acceptor beads and biotin-labeled antibodies were mixed in 20 μl, after which 5 μl sample suspension or rotavirus antigen was added. The 25 μl mixture was incubated at 37°C for 15 min, and then 25 μl of streptavidin donor beads were added. After incubation for an additional 10 min at 37°C, the signal was read by SpectraMax™ I3 (Molecular Devices, Sunnyvale, CA, USA).

To optimize the concentration of biotinylated antibodies, antibodies labeled acceptor beads, and streptavidin-coated donor beads, rotavirus antigen at low, middle or high concentrations were detected with three replicates per group. Signal to noise (S/N) ratios were calculated. To evaluate the sensitivity of AlphaLISA, we first performed a 1:25-fold dilution of rotavirus antigens and then prepared serial 5-fold dilutions for AlphaLISA detection. The cutoff value was defined as the average fluorescence intensity of the negative control group plus three standard deviations.

Repeatability of AlphaLISA was assessed by tests of two levels of antigen concentrations. Intra-assay variation was calculated from the variation of 12 determinations of low and high antigen concentrations (1:5^7^ and 1:5^3^ dilution ratio) on the same plate and in the same test. On the other hand, inter-assay variation was calculated by antigen detecting in the same manner once-a-day for three consecutive days. The average measured value, standard deviation (SD), and coefficients of variation (CV) were calculated.

### RT-qPCR test

Since most infection from human rotavirus is caused by group A viruses, group A rotavirus nonstructural protein 3 gene was tested by RT-qPCR. The total RNA of stool samples was extracted using a QIAamp Viral RNA Mini Kit (Qiagen, Valencia, CA, USA). RT-qPCR was performed for each sample using AgPath-ID™ one-step RT-PCR reagents (Thermo Scientific, Waltham, MA, USA). The primers and probe used for detecting human group A rotavirus (Rota-F: ACCATCTWCACRTRACCCTCTATGA; Rota-R: GGTCACATAACGCCCCTATAGC; Rota-P: AGTTAAAAGCTAACACTGTCAAA) (13) were synthesized by Sangon Biotech (Shanghai) Co., Ltd. The reaction was performed on a ViiA™ 7 real-time PCR system (Applied Biosystems, CA, USA). The cycling conditions were 45°C for 10 min, 95°C for 10 min, and 40 cycles of 95°C for 15 s and 60°C for 45 s. The result was considered positive when the cycle threshold (Ct) value was ≤ 38 and was considered negative when it was > 38. Fourteen stool samples were re-tested using Rotavirus (Group A) Nucleic Acid Assay Kit (Shanghai Liferiver Biotechnology Co., Ltd.), and reverse transcription, amplification, and detection were conducted according to the manufacturer's instructions.

### ICA test

A Rotavirus Antigen Assay Kit (Guangzhou Wondfo Biotechnology Co., Ltd.) was used for the immunochromatographic assay. Ten milligram or 50 μl of stool samples were collected and mixed with the sample diluent. Two to three drops (about 60 μl to 80 μl) of the sample solution were added to the sample loading area. After 10 min, the result was considered positive if one line was observed in the control area (C) and another line was observed in the test area (T).

### Statistical analysis

Data are presented as mean ± SD. The student's *t*-test was used to compare two groups. The ROC curve, area under the ROC curve (AUC), cutoff value, and kappa coefficient were calculated using the statistical analysis software SPSS 22.0. *P* < 0.05 was considered statistically significant.

## Results

### Optimal concentration of biotinylated antibodies, acceptor-conjugated antibody beads, and streptavidin-coated donor beads

**Figure 1:**
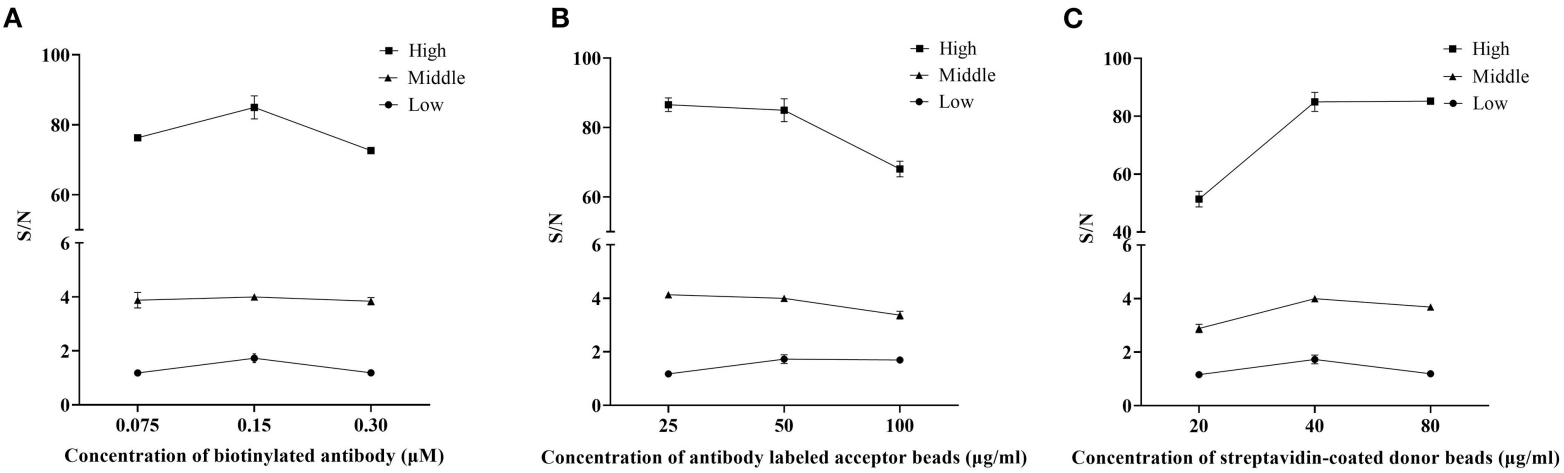
Optimization of the AlphaLISA experiment. The effect of a single variable on AlphaLISA S/N was examined by making the other factors constant. **(A)** Effect of the concentration of biotinylated antibodies (μM); **(B)** Effect of the concentration of antibody labeled acceptor beads (μg/ml); **(C)** Effect of the concentration of streptavidin-coated donor beads (μg/ml). Low: the low concentration of rotavirus antigen (dilution ratio: 5^−7^); Middle: the middle concentration of rotavirus antigen (dilution ratio: 5^−5^); High: the high concentration of rotavirus antigen (dilution ratio: 5^−3^). Mean values from 3 trials are plotted, with error bars denoting the standard deviation.

Optimal concentrations of biotinylated antibodies would be determined first. The concentration of the biotinylated antibodies varied (0.075, 0.15, and 0.30 μM) while we kept the acceptor-conjugated antibody beads and streptavidin-coated donor beads constant to ensure optimal performance when detecting rotavirus. Results of this assay (Figure 1A) showed that using 0.15 μM of biotinylated antibodies produced the largest S/N ratios, regardless of the antigen concentration. Therefore, 0.15 μM was chosen for subsequent AlphaLISA assay.

The concentration of acceptor beads and streptavidin-coated donor beads was essential for the immunoassay sensitivity and linear range. On the one hand, an excessive amount of chemibeads would provide more opportunities for random collisions between the acceptor beads and donor beads, which increases the background signal and decreases sensitivity. On the other hand, an extremely low amount of chemibeads would decrease the signal and affect the sensitivity of the analysis (14). Therefore, in this assay, 25, 50, and 100 μg/ml antibodies labeled acceptor beads and 20, 40, and 80 μg/ml streptavidin-coated donor beads were separately tested to determine the optimum concentration for the AlphaLISA experiment. After considering both S/N and sensitivity, the optimal concentration of acceptor beads was 50 μg/ml (Figure 1B). Similarly, the optimal concentration of streptavidin-coated donor beads was 40 μg/ml (Figure 1C).

### Sensitivity and repeatability of detection

**Figure 2:**
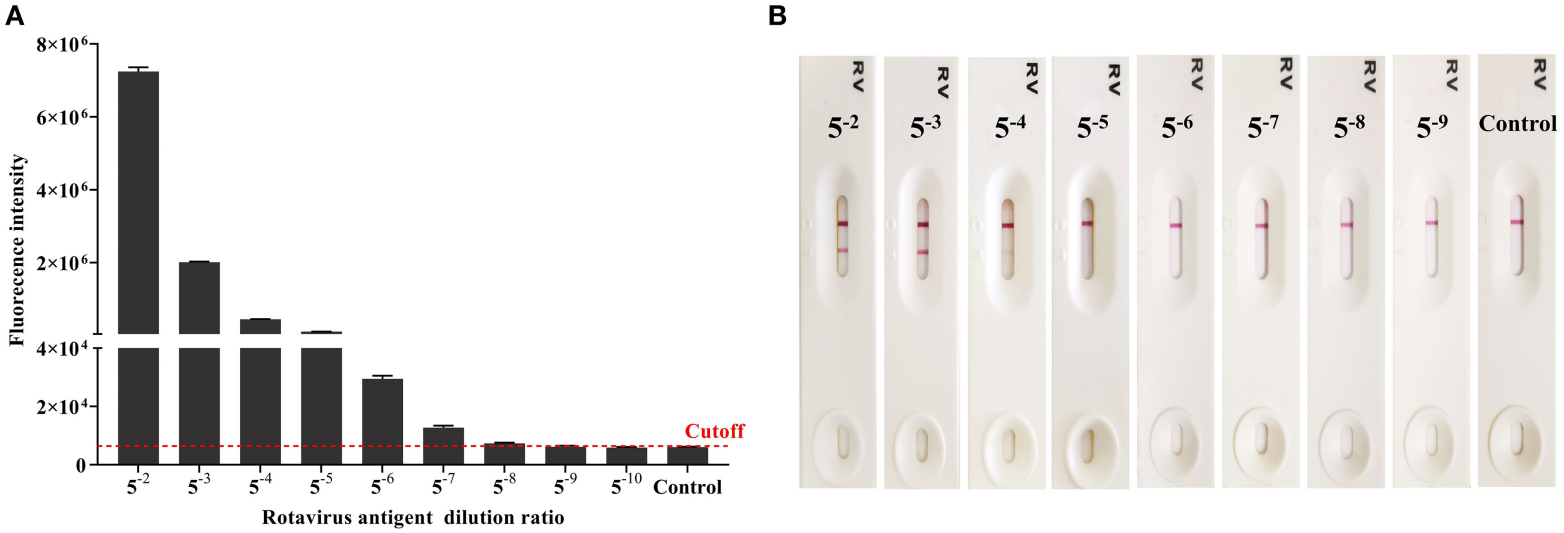
Comparison of the detection sensitivity for AlphaLISA and ICA (Result of one experiment shown only, see Supplementary Figure 1 for the others). **(A)** Rotavirus antigen detection using AlphaLISA. Mean values from 3 trials are plotted with error bars denoting the standard deviation; **(B)** Rotavirus antigen detection using the ICA.

Rotavirus antigen was tested using AlphaLISA and ICA, to compared the sensitivity of the two methods. AlphaLISA could detect rotavirus antigens at a dilution of 1: 5^8^, whereas ICA could only detect rotavirus antigens in 1: 5^4^ dilutions (Figure 2). Therefore, the sensitivity of AlphaLISA significantly exceeded that of ICA. The repeatability experiment showed that, for AlphaLISA, the intra-assay CV was 2.99-3.85%, and the inter-assay CV was 5.27-6.51% (Table 1). This indicates that the AlphaLISA had sufficient repeatability.

**Table 1:** Intra- and inter-assay coefficients of variation (CV).

**Antigen dilution ratio**	**Intra-assay (*****n*** = **12)**		**Inter-assay (*****n*** = **36)**	
	**Mean ±SD**	**CV (%)**	**Mean ±SD**	**CV (%)**
5^−7^	12805.25 ± 493.48	3.85	12253.03 ± 797.85	6.51
5^−3^	1930305.08 ± 57758.57	2.99	1884488.69 ± 99378.86	5.27

### Optimization of sample pretreatment conditions

**Figure 3:**
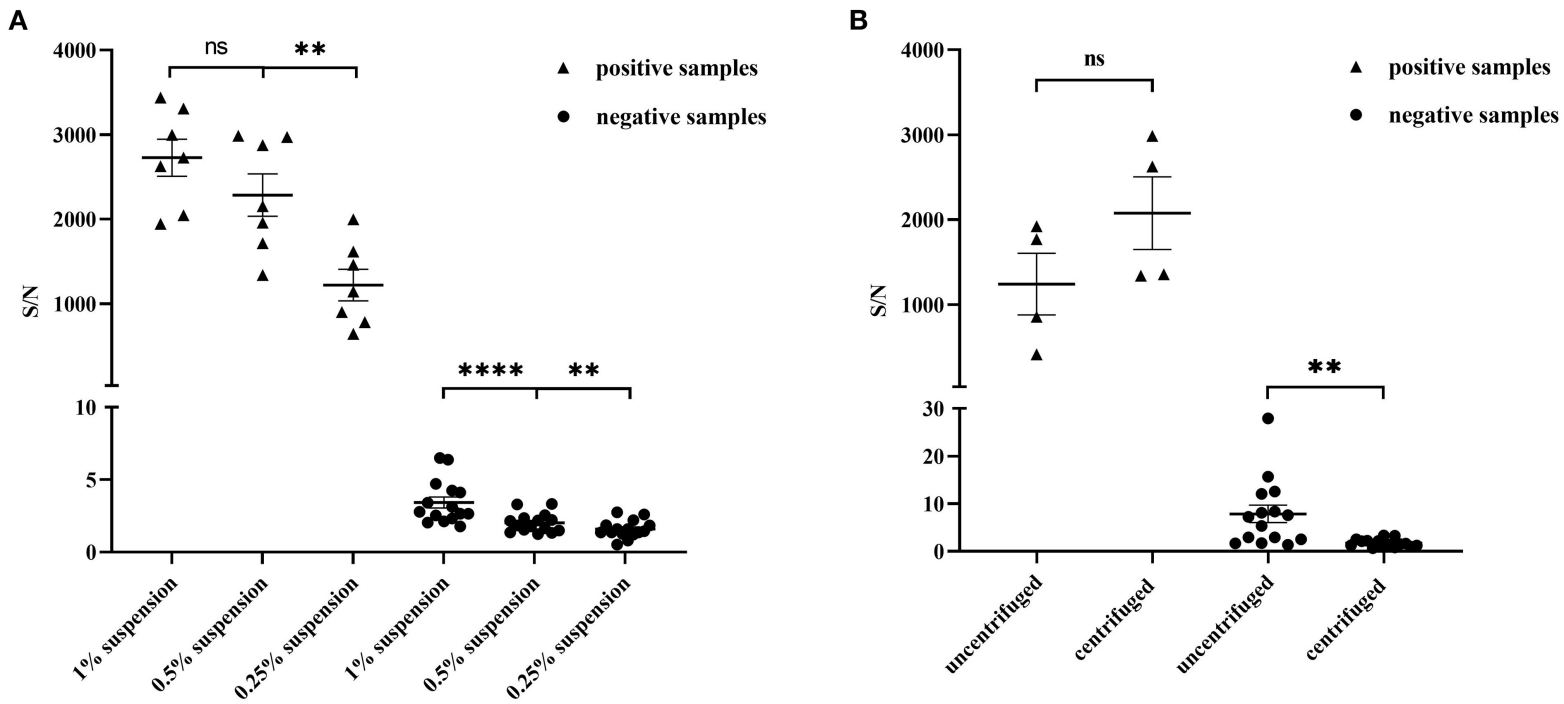
Optimization of the stool sample pretreatment for AlphaLISA experiment. **(A)** Influence of 1% suspension, 0.5% suspension and 0.25% suspension on the test results of positive stool samples (*n* = 7) and negative stool samples (*n* = 15); **(B)** Influence of uncentrifuged and centrifuged for positive stool samples (*n* = 4) and negative stool samples (*n* = 15) on detection; **P < 0.01; ****P < 0.0001; ns, not significant.

Due to the complexity and heterogeneity of the stool samples, they often must be pretreated to remove interfering substances that might be present. We prepared sample suspensions at different concentrations (1, 0.5, and 0.25%) to evaluate the influence of sample dilution on detection. To avoid false-positive results and ensure a high S/N ratio in the positive samples, the optimal concentration of suspension was 0.5% (Figure 3A). We then evaluated the influence of centrifugation treatment on detection and found that it had little effect on the positive samples (Figure 3B). However, for negative samples, the S/N ratio significantly decreased after centrifugation. The treatment reduced the occurrence of false-positive results. The stool samples were diluted into 0.5% (w/v) suspension in the subsequent experiments and centrifuged.

### Optimal cutoff value

**Figure 4:**
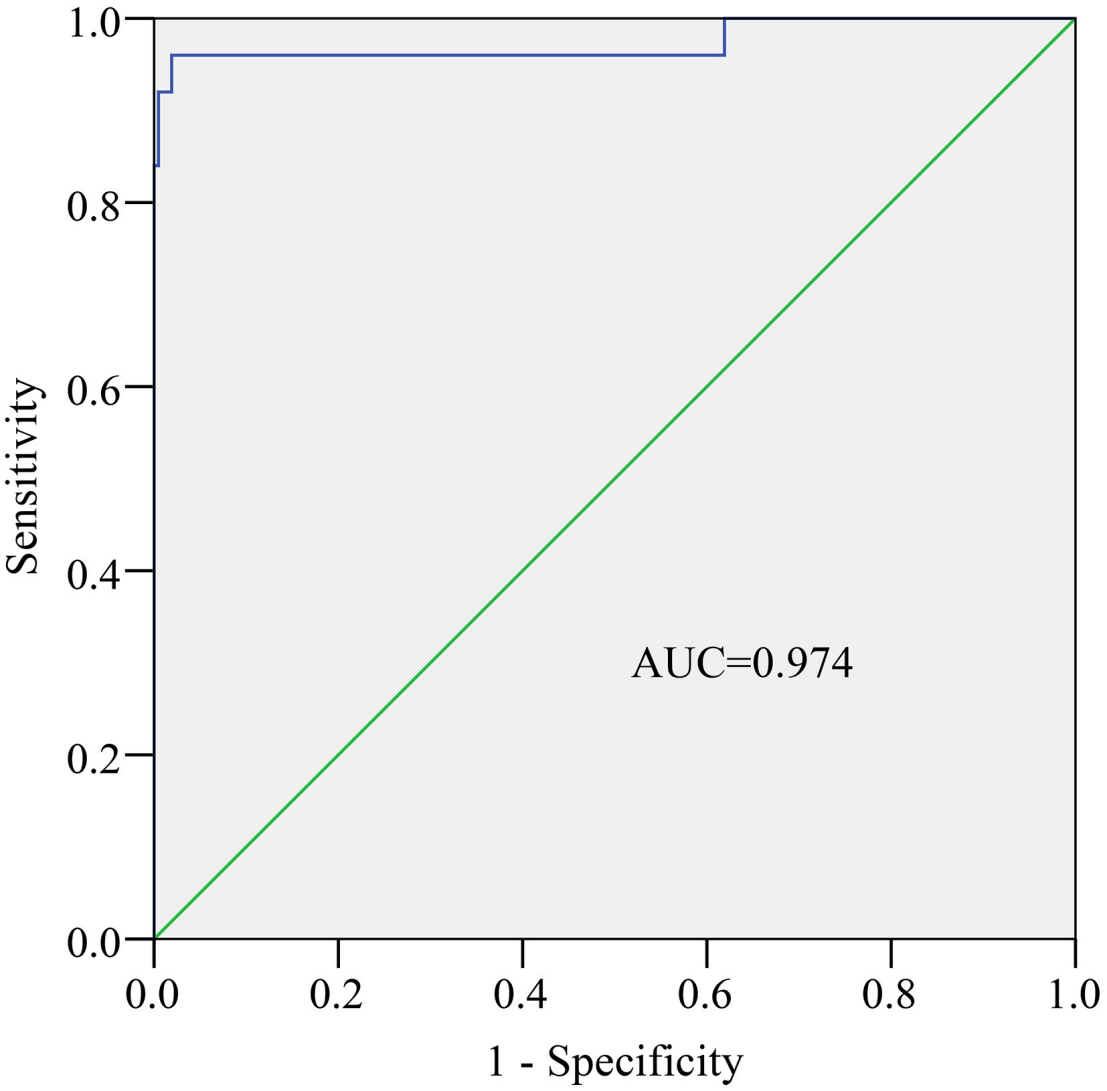
Receiver operating characteristic (ROC) curve of AlphaLISA. AUC, area under the curve.

ROC was plotted based on 235 stool samples, and RT-qPCR is considered the gold standard for detecting rotavirus. The AUC was 0.974 (*P* < 0.001, Figure 4). An AUC > 0.95 typically indicates a very high diagnostic value for a test. Therefore, we chose a cutoff value of 4.9142 on the ROC based on the optimal sensitivity and specificity (Figure 4). Samples with S/N ratio ≥ 4.9142 are considered rotavirus positive, while samples with S/N ratio < 4.9142 are considered rotavirus negative.

### Cross-reactivity of AlphaLISA

**Figure 5:**
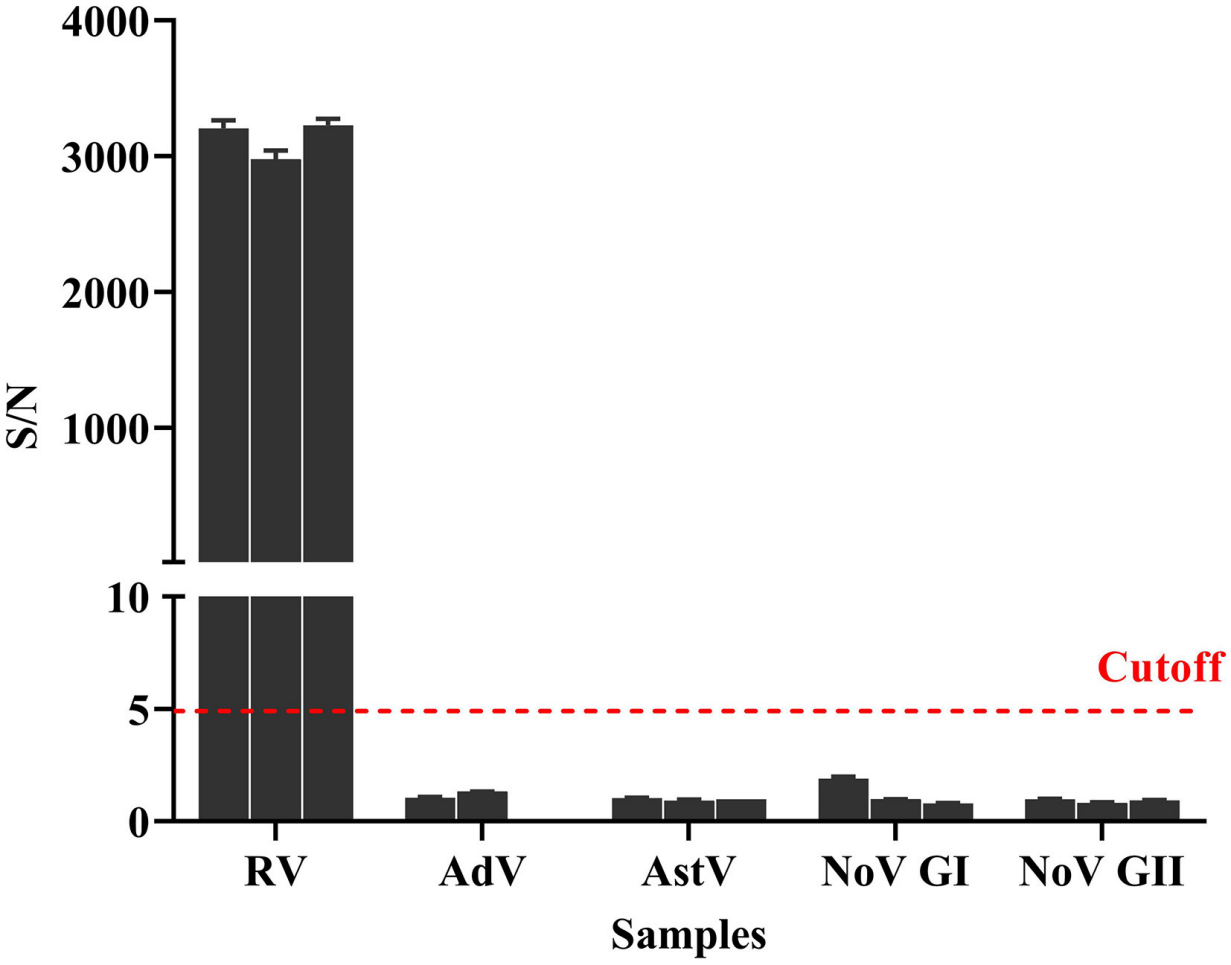
Cross-reaction of AlphaLISA. Different diarrhea virus-positive samples (3 for RV, 2 for enteric AdV, 3 for AstV, 3 for NoV GI, and 3 for NoV GII) were analyzed with AlphaLISA. The bar represents the standard deviation. The dotted line represents the cutoff value. RV, rotavirus; AdV, adenovirus; AstV, astrovirus; NoV GI, norovirus genogroup I; NoV GII, norovirus genogroup II.

In addition to rotavirus, other viruses can cause acute gastroenteritis (15–17). For the cross-reactivity evaluation of the detection method used in this study, stool samples with rotavirus, adenovirus, astrovirus, norovirus genogroup I, and norovirus genogroup II were tested using the AlphaLISA method. Triplicate samples and negative controls were set in this assay, and the cutoff value was used to classify them as negative or positive. Results (Figure 5) showed that AlphaLISA could accurately distinguish rotavirus from other diarrhea viruses.

### Comparison of AlphaLISA, ICA, and RT-qPCR for detecting rotavirus in clinical stool samples

Two hundred and thirty five stool samples were tested using AlphaLISA, RT-qPCR, and ICA. The comparative results of AlphaLISA, ICA, and RT-qPCR for detecting rotavirus were shown in Table 2. The overall agreement rates between the three methods were 94.04% for rotaviruses. The 14 samples with discordant results in 3 methods were reconfirmed with the commercial PCR kit, and all were consistent with the RT-qPCR results. The overall agreement, positive agreement, and negative agreement of AlphaLISA compared with RT-qPCR were 97.87, 96.00, and 98.10%, respectively. The weighted kappa coefficient was 0.894, and the asymptotic 95% confidence interval was 0.802–0.986. The overall agreement, positive agreement, and negative agreement of ICA compared with RT-qPCR were 95.74, 84.00, and 97.14%, respectively. The weighted kappa coefficient was 0.784, and the asymptotic 95% confidence interval was 0.655–0.913.

**Table 2:** Comparison of rotavirus detection results by AlphaLISA, ICA, and RT-qPCR.

**Virus**		**RT-qPCR**			
		**Positive agreement**	**Negative agreement**	**Total agreement**	**Kappa coefficient (95% CI)**
Rotavirus (94.04%, 221/235)^*^	AlphaLISA	96.00% (24/25)	98.10% (206/210)	97.87% (230/235)	0.894(0.802–0.986)
	ICA	84.00% (21/25)	97.14% (204/210)	95.74% (225/235)	0.784(0.655–0.913)

* Overall agreement rate among three assays.

CI, confidence interval.

Comparison of the AlphaLISA and RT-qPCR tests showed that the results agreed with each other (Kappa > 0.75). However, AlphaLISA yielded four false-positive results (for which RT-qPCR and ICA yielded negative results). This suggested that AlphaLISA might provide false-positive results. One stool sample showed negative results using the AlphaLISA and ICA methods but positive results using RT-qPCR. The Ct value of this sample was 36.89 (Table 3). A low viral load in this sample could be the reason for negative results with AlphaLISA and ICA. In contrast, ICA yielded six false-positive results (RT-qPCR and AlphaLISA yielded negative results) and four false-negative results compared to RT-qPCR, showing that AlphaLISA was more accurate than routine ICA methods for rotavirus detection. Additionally, we found that AlphaLISA was less consistent with ICA, with the weighted kappa coefficient of 0.732 and the asymptotic 95% confidence interval of 0.593-0.871. The overall agreement, positive agreement, and negative agreement of AlphaLISA compared with ICA were 94.47, 77.78, and 96.63%, respectively (Supplementary Table 1).

**Table 3:** The test results of rotavirus-positive stool samples.

	**RT-qPCR**		**AlphaLISA**		**ICA**	
**Sample number**	**Ct**	**Results**	**S/N**	**Results**	**Picture**	**Results**
1	16.50	P	2972.18	P	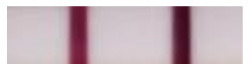	P
2	16.52	P	3228.74	P	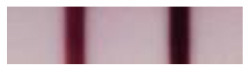	P
3	17.14	P	3349.40	P	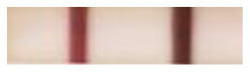	P
4	17.69	P	2624.43	P	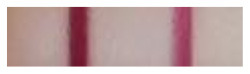	P
5	19.5	P	2874.82	P	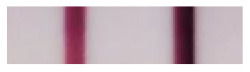	P
6	19.88	P	1356.93	P	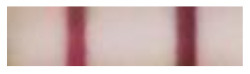	P
7	19.94	P	2152.41	P	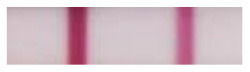	P
8	20.95	P	1957.64	P	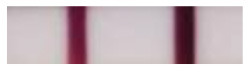	P
9	21.22	P	1338.63	P	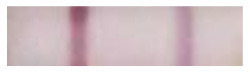	P
10	21.48	P	2985.35	P	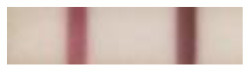	P
11	21.80	P	1714.18	P	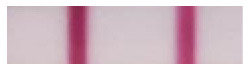	P
12	21.97	P	3183.47	P	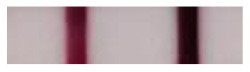	P
13	22.86	P	2978.86	P	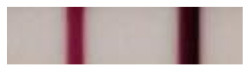	P
14	23.13	P	3206.75	P	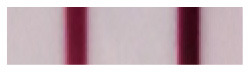	P
15	25.91	P	998.82	P	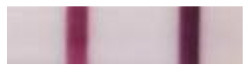	P
16	25.93	P	1011.61	P	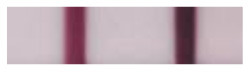	P
17	31.73	P	7.49	P	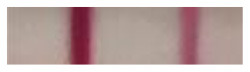	P
18	32.28	P	203.46	P	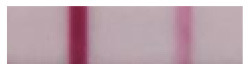	P
19	32.71	P	161.93	P	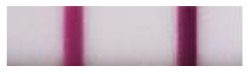	P
20	33.01	P	140.19	P	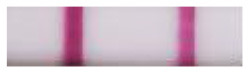	P
21	33.18	P	39.09	P	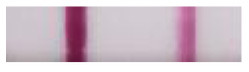	P
22	34.68	P	14.38	P	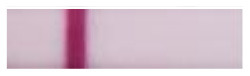	N
23	35.06	P	5.14	P	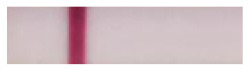	N
24	35.98	P	7.31	P	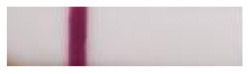	N
25	36.89	P	1.03	N	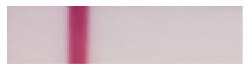	N

P, Positive; N, Negative.

## Discussion

Rotaviruses is the major pathogen cause of acute viral gastroenteritis in infants and young children worldwide, producing a significant disease burden. There is also an extensive literature linking rotavirus to gastroenteritis in adults throughout the world (18). Acute gastroenteritis due to rotavirus can lead to vomiting and watery diarrhea, which in turn causes body fluid loss leading to dehydration and affects patient's quality of life seriously. Although the rotavirus incidence has dramatically declined with vaccination among high-income countries, the number of diarrhea and rotavirus deaths remains high in low-income populations with poor access to safe water, sanitation, and urgent medical care in developing countries (1–3). Hence, in order to enable rapid confirmation of acute gastroenteritis pathogens, a rapid and sensitive detection method is still a major concern for monitoring rotavirus outbreaks.

AlphaLISA is a homogeneous immunoassay with high sensitivity which have no need for any separation/washing steps but 5 μl sample and a two-step mixed reaction for detection (19–21). Currently, AlphaLISA has been used for the detection of a wide variety of analytes from proteins to peptides and other small molecules. And it has been extensively used for the detection of several infectious viruses, namely, Hepatitis B virus in human serum and African swine fever virus in porcine serum with high sensitivity and specificity (19, 22). In this study, AlphaLISA method was developed for rapid and sensitive detection of rotavirus.

Numerous AlphaLISA assays have been reported in a variety of sample types ranging from cell lysates (23, 24), to serum (25, 26), to food (12, 27). In this study, we showed, for the first time, the applicability of the AlphaLISA technology for the detection of stool samples. The stool suspension samples were pretreated by dilution and centrifugation to remove interfering substances that might be present, and non-specific reactions of the AlphaLISA assay had been greatly reduced. The AlphaLISA method could detect rotavirus well in stool samples.

The sensitivity and specificity of AlphaLISA in detecting rotavirus was lower than the RT-qPCR method, since the molecular biological methods were considered more sensitive than the immunological method and the RT-qPCR test is considered a gold standard for detecting diarrhea viruses (28, 29). For patients with acute gastroenteritis, the RT-qPCR method is time-consuming as it requires 3 to 4 h to conduct and get the test results. AlphaLISA was a rapid and homogeneous immunoassay which could test rotavirus in 30 min. It could be used as a novel potential on-site rapid detection method and showed better consistency with RT-qPCR than routine ICA methods for rotavirus detection. Compared to convenient operation of ICA, AlphaLISA method still requires manual operation and specific laboratory instruments. But AlphaLISA method is performed according to simple “mix-and-measure” protocols, which is ideally suited for miniaturization and automation. Miniaturization, automated instrumentation will enable this method to be used for point-of-care-testing (POCT) of rotavirus infection. In addition, the method can be used to test up to 384 samples simultaneously by using 384-well plates to increase throughput, reduce reagent consumption. Using portable instruments and reducing reagent costs will facilitate the commercialization and wide application of AlphaLISA for rotavirus detection. Before that larger-scale and multicenter clinical specimens test should be conducted to further validate the commercial utility of AlphaLISA.

In conclusion, the AlphaLISA method developed in this study have high sensitivity and specificity in detection of rotavirus, with short turnaround time (30 min), high reproducibility, and high consistence of detection results to the RT-qPCR method. Therefore, AlphaLISA could be a useful screening tool for rapidly and accurately diagnosing rotavirus infection during viral outbreaks.

## Data availability statement

The original contributions presented in the study are included in the article/Supplementary material, further inquiries can be directed to the corresponding author/s.

## Ethics statement

The studies involving human participants were reviewed and approved by the Institutional Review Board (IRB) of the Fifth Medical Center of PLA General Hospital (reference no. ky-2019-1-4) and Dongfang Hospital (reference no. JDF-IRB-2020003501). Written informed consent to participate in this study was provided by the participants' legal guardian/next of kin.

## Author contributions

PL, QL, and HJ conceived and designed the study and participated in the review and editing of the manuscript. YW and YZ carried out the experimental work and analyzed the data. YW, QL, and HJ wrote the original draft of the manuscript. All authors participated in interpreting the results, read, and approved the final version of this manuscript.

## Funding

This work was supported by research grants from the Chinese State Key Project Specialized for Infectious Disease (Grant No. 2018ZX10711001-003-001).

## Conflict of interest

The authors declare that the research was conducted in the absence of any commercial or financial relationships that could be construed as a potential conflict of interest.

## Publisher's note

All claims expressed in this article are solely those of the authors and do not necessarily represent those of their affiliated organizations, or those of the publisher, the editors and the reviewers. Any product that may be evaluated in this article, or claim that may be made by its manufacturer, is not guaranteed or endorsed by the publisher.
