# Supplementary material for: Development of a rapid homogeneous immunoassay for detection of rotavirus in stool samples
Source: Front Public Health. 2022 Aug 4;10:975720. doi: 10.3389/fpubh.2022.975720 (PMC9386352; doi:10.3389/fpubh.2022.975720)
Supplement: Supplementary file 1 [file Table_1.DOCX]

**Supplementary Table 1**

Comparison of rotavirus detection results by AlphaLISA and ICA.

|  | | ICA | | | | Total | |  |
| --- | --- | --- | --- | --- | --- | --- | --- | --- |
|  |  | Negative | | Positive | |  |  |  |
| AlphaLISA | Negative | | 201 | | 6 | | 207 | |
|  | Positive | | 7 | | 21 | | 28 | |
|  | Total | | 208 | | 27 | | 235 | |
|  | agreement  rate | | 96.63% | | 77.78% | | 94.47% | |

Kappa coefficient (95% confidence interval): 0.732 (0.593, 0.871).
