# Supplementary figures and images for: Development of a rapid homogeneous immunoassay for detection of rotavirus in stool samples
Source: Front Public Health. 2022 Aug 4;10:975720. doi: 10.3389/fpubh.2022.975720 (PMC9386352; doi:10.3389/fpubh.2022.975720)

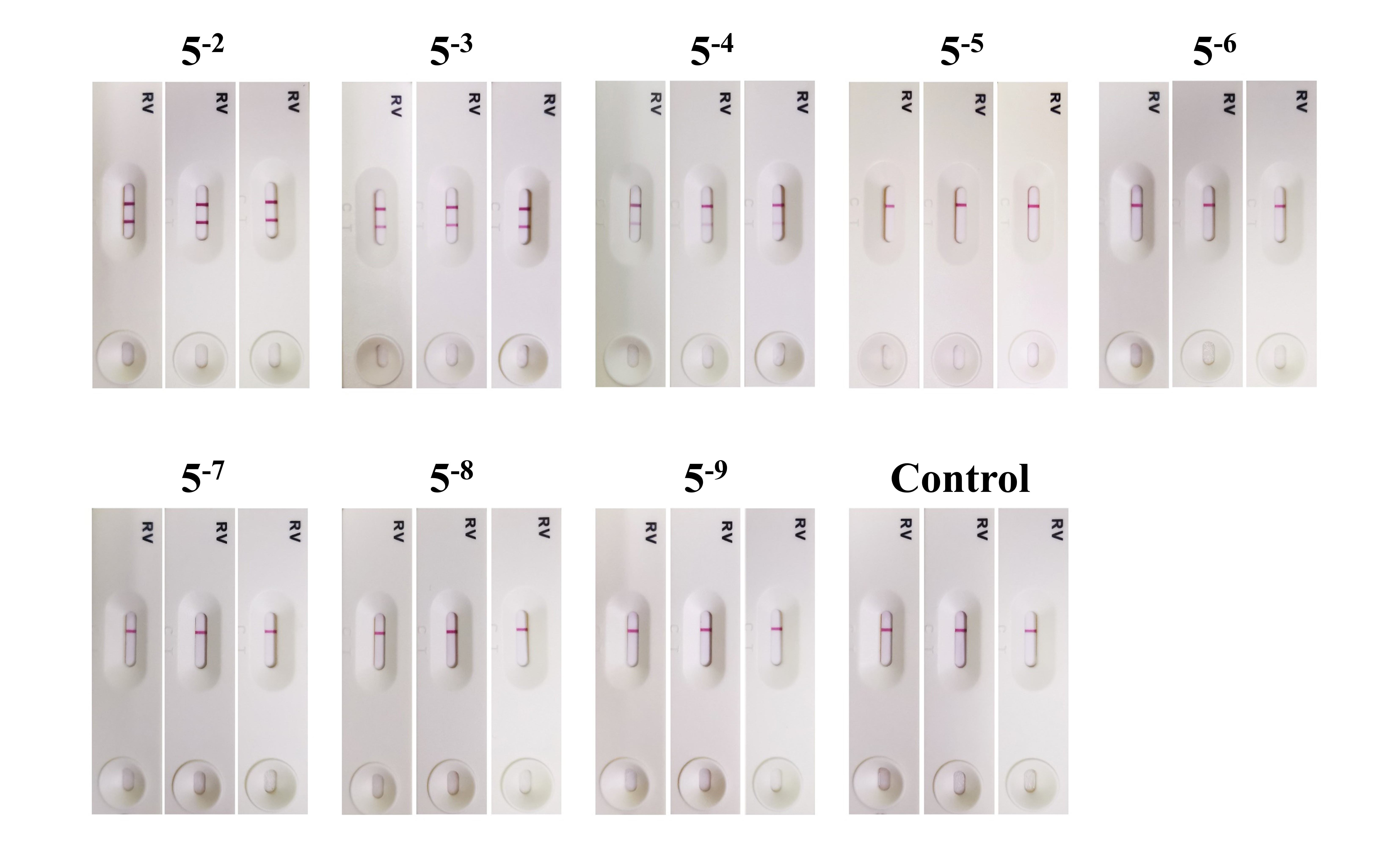

Supplement: Supplementary file 2 [file Image_1.JPEG]
